# Transforming planning and policy making processes at the intersections of climate, equity, and decolonization challenges

**DOI:** 10.1038/s42949-023-00126-9

**Published:** 2023-08-03

**Authors:** Lindsay Cole, Maggie Low

**Affiliations:** 1 School of Community and Regional Planning, The University of British Columbia, Vancouver, British Columbia Canada; 2 Faculty of Graduate Studies, Emily Carr University of Art + Design, Vancouver, British Columbia Canada

**Keywords:** Environmental studies, Energy justice, Sustainability, Climate-change policy

## Abstract

Cities are facing increasing pressures to address complex challenges of climate change, equity, and reconciliation with Indigenous Peoples as intersecting issues, and innovation into planning and policy-making processes is urgently needed to achieve this. It is no longer good enough to work on these challenges discreetly, or solely within the dominant, western colonial paradigm and practices of governance. There are ongoing harms being caused by climate work that does not embed justice, and there are missed opportunities for synergies across these domains as they have the same systemic root causes. Cities must adapt and transform the processes and practices of planning and policy-making in order to work at these problematic roots. Drawing on an empirical study, this article describes how social innovation, systemic design, and decolonizing practices can shape a different approach to planning and policy-making processes when working at the intersections of climate, equity, and decolonization.

## Introduction

Over the last decade, the possibilities and pressures that cities are facing related to complex challenges like climate change have surged. The global migration of people into cities has continued, and organizations like the International Panel on Climate Change have begun emphasizing the vital role of sub-state actors in climate adaptation and mitigation in actions related to land-use planning, infrastructure, transportation, housing, community development, and others^1^. National and international network-serving and advocacy organizations working on climate change and other sustainability issues at the municipal level have emerged, for example, C40, the Global Covenant of Mayors, the Carbon Neutral Cities Alliance, and the Urban Sustainability Directors Network. Political and civil servant leadership from cities is a powerful force for change on the world stage, with growing influence on other levels of government, business, civil society, and the public imagination in far-reaching ways.

While city leadership on climate-related work continues to strengthen, it is often treated as a technical, engineering, land use, and financial challenge and the related systemic challenge of ongoing and growing inequity tends to be un(der) addressed in urban climate plans and policies^2–4^. While cities work to mitigate and adapt to a changing climate, the very same people, lands, and waters who have been systemically excluded, oppressed, and exploited by the dominant structures and processes of government continue to be left out^5–7^. This is a compelling impetus for transformative innovation, so that in their eagerness to act on climate, cities do not recreate and perpetuate problematic practices from the past/present that result in inequity and oppression^8,9^. There is an opportunity to reimagine the policy-making and planning processes of local government to ensure that climate-related concerns are considered in an integrated way with equity, justice, reconciliation, and decolonization work^10,11^. Climate change issues have the same systemic root causes of colonialism, capitalism, white supremacy, patriarchy, ableism, and cis-heteronormativy and can and should be worked on together^12–14^.

The standard policy-making and planning processes used in local governments in many parts of the world, and embedded in the New Public Management, western, colonial governance paradigms, are being reinforced rather than being reimagined in many cases as governments work to innovate in response to complex challenges like climate change^15–21^. This remains the case even as the suitability of these paradigms to respond to complex, social and ecological justice challenges at the rate, scale, and depth required is being questioned, with calls for transformation and innovation becoming louder and more urgent^22–24^. The standard policy making cycle of agenda-setting, policy formulation, decision making, policy implementation, and policy evaluation is deeply embedded into the thinking, structures, and processes of governments around the world. This cycle “offers practitioners a means to conceptually and practically guide an issue methodically through the policy process”^25^ and has been both described and critiqued for decades, with academics seeking further nuance and complexity to the model and practitioners requiring a variety of process interventions that work in the real world on increasingly volatile, uncertain, complex, and ambiguous challenges^26^.

Similar to the standard policy making cycle, urban planning is characterized by evidence-based rationality and objectivity, established administrative routines, rule-bound bureaucratic procedures, and significant emphasis on a planner’s ability to know what is good for people generally, or the public interest^17,27,28^. While planning has evolved over decades to consciously seek to redistribute power through equity planning^29^ and to better align with the goals of communities through radical planning, planning processes often still involve “the analysis of problems, the setting of broad objectives, the survey of available resources, and the establishment of specific operating targets''^28^. As planning processes continue to impede innovation and adaptation to dynamic pressures, planning must evolve to address the unpredictability of the complex challenges we face^27^. For instance, a pressing challenge for planning (and policy making) is reconciling its role in perpetuating the dispossession, oppression, and marginalization of Indigenous Peoples and communities and grappling with the reality that all planning in Canadian cities happens on stolen Indigenous lands^30–32^.

In Decolonizing Methodologies, Linda Tuhiwai Smith provides a working definition of decolonization, which “does not mean and has not meant a total rejection of all theory or research or Western knowledge. Rather it is about centring [our] concerns and world views and then coming to know and understand theory and research from our own perspectives and purposes”^33^. Central to decolonizing methodologies within the context of working to transform complex urban climate, equity, and decolonization challenges is recognizing the coexistence of distinct knowledge systems, and how these distinct knowledge systems can work alongside each other in a good way^34^. Conventional systems-based approaches have foundations in the scientific method that pursues knowledge in an analytical way, while Indigenous ways of knowing is about (for Elders) the pursuit of wisdom in action^35^ and using the mind, body, emotion, and spirit to make meaning of the world around us^36^. Importantly, Indigenous ways of knowing are innately tied to the land^37,38^. Scholars and practitioners are applying decolonizing principles to their work, and central to this process is the idea that change happens from people going inward within themselves^34^. Planning practice is starting to acknowledge this turn inwards as well, and is recognizing that people are not disconnected from other living beings and that we must connect our present lives with nature^39^. These principles are a departure from the rational and objective mindsets that planners and policymakers have been trained to use for decades.

Concepts like justice, equity, and decolonization are not static, singular, with clear and agreed upon meanings. They are contested and actively being worked out in institutions, processes, and everyday life^40,41^ and are expressed differently according to place, context, history, and culture. Tuck and Yang (2012, p.7) point out that settler colonialism and its decolonization implicates and unsettles everyone and argue that decolonization is only about land and Indigenous life^42^. In other words, decolonization is *not* a metaphor for “other things we want to do to improve our societies” (p.1)^42^. This article pushes us to think critically about the word decolonization and what it means in planning and policy making practice. Tuck and Yang’s settler moves to innocence reveal attempts to reconcile settler guilt and complicity that do not require giving up land, power, or privilege. We agree the central work of decolonization should be the return of land. At the same time, as planners and policymakers doing research entangled with practice, we are motivated to find meaningful entry points to decolonization through practices that every planner and policymaker can enact. In this research we take the perspective that settler colonialism is an ongoing structure and not an event^43^. Our guiding principle, at least for now, is that decolonization work is for all of us to do and so we are enacting it as practice(s), and expressing these practices in multiple and embodied ways in the specific context of complex policy making and planning challenges. That said, decolonization work will look different for non-Indigenous peoples than it does for Indigenous Peoples. For Bob Joseph (2023), acts of decolonization restore Indigenous worldviews, restore culture and traditional ways, and decolonization replaces Western interpretations of history with Indigenous perspectives of history^44^. Here we focus on policy-making and planning practice happening within settler colonial systems of local government, and explore what and how we might (un)learn and practice equity, justice, and decolonization for/among each other in the context of urban climate work.

The nature of planning and policy-making processes as they relate specifically to climate change mitigation and adaptation challenges are distinct and complex. Climate change research has rapidly expanded and evolved over the last decade, with studies being undertaken in every discipline, using diverse methodologies, providing insights for every sector, and in every part of the world. The collective of researchers that forms the Intergovernmental Panel on Climate Change (IPCC) is capturing this vast field and then informing, motivating, and compelling responses from global actors through their Working Group reports and summaries for policy makers^45^. With each report of the IPCC there is stronger consensus about the impacts, extent, and urgency of a changing climate. Promising pathways for action are becoming clearer and better evidenced, and enabling conditions and barriers to action are descriptive and explicit. The processes of activating and scaling robust, global responses by elected leaders, civil servants, financial institutions, businesses, nonprofits, in the (now) short time frames to reduce the most significant impacts on our already changing world remains fraught and inconsistent. There is much to gain and much to lose, with unequal distribution of burdens and benefits. There is an unreliable patchwork of country- and sub-state level policies, plans, targets, and financing commitments with varying degrees of commitment and impact, and very few enforceable accountability mechanisms in place. This approach continues to leave those most vulnerable to a changing climate improperly resourced to act^46^. The roles of sub-state actors, including cities, is becoming more visible in this recent IPCC work, and this global climate context plays out in the daily lived experiences of city dwellers. There are many tensions and conflicts that exist in urban climate policy and planning, with civil servants needing to navigate the complexities of varying political support, limited jurisdiction, inadequate financing tools, challenges with data availability and staff expertise, bureaucratic silos, and inconsistent regulatory and accountability frameworks.

What then is demanded/required of urban planners and policymakers if the dominant approaches taken in their work no longer serve complex, intersectional, and systemic challenges of climate, equity, and decolonization? Theories and practices from the fields of social innovation, systemic design, and decolonizing methodologies (see above) offer promising alternatives for working differently on these complex challenges. Social innovation theory integrates many fields of social science and systems practice in service of shifting deep structures - hearts, minds, and culture - and of scaling and movement building for transformative socio-ecological change. Systemic design brings a designerly mindset, lineage, frame of reference, training, approach, and set of experiences into this mix. The experimentation- and action-as-learning bias of systemic design ensures that innovations land in the real world, in testable experiences, with- and for the people and places most affected by a particular challenge. Decolonizing methodologies ensures that social innovations and systemic design do not work within dominant and problematic paradigms of colonization and oppression and inadvertently perpetuate these systems under the guise of ‘innovation.’ Instead wisdom and insight is sourced from culture, history, people, and possibility and is deeply grounded in place-based and relational practice. Together they can inform the evolution of policy making and planning processes in ways that may result in more significant shifts than are possible when working within dominant systems that have oppression, inequity, and exploitation of humans and nature baked in.

Social innovations work to transform the behaviours, structures, mindsets, and beliefs of a social system with an intent to more skillfully and effectively respond to problems than is possible through existing or commonly used approaches that are less likely to address root causes of wicked challenges^47–49^. An innovation is ‘social’ in that it aims to shift social practices, ideas, beliefs, interests, power, and agency so that innovations are diffused, scaled, institutionalized, or otherwise integrated and made routine. Social innovation processes follow a series of stages, often facilitated as iterations or cycles of experimentation and learning as new/different solutions emerge through a collaborative process, including the following stages: problem/challenge framing; action or user research; generating ideas; developing and testing ideas through experimentation; evaluation, reframing, and iteration; implementation at increasing levels of fidelity; scaling successful solutions out/up/deep; and ultimately changing systems^50–54^. Some social innovation process archetypes stay focused on the specific challenge they are tackling, whereas others bring in a stronger personal transformation orientation, recognizing that inner work is required for outward-oriented change^55–59^.

Systemic design integrates systems thinking into human-centered and service design approaches for working creatively on complex challenges. Jones & Kijima (2018) say that “systemic design advances an integrative interdiscipline with the potential to implement systems theory with creative methods and mindsets, by bringing deep technical knowledge, aesthetic skill, and creative implementation to the most abstract programmes of collective action” (p. ix)^60^. Systemic design processes tend to oscillate between convergent and divergent thinking and doing, hold a strong bias toward action and experimentation, and enable right-sized risk-taking and micro-failures early on as critical pathways for learning. Examples of systemic design process archetypes include: ask, try, do; strategy, discover, design, develop, deploy; and inquire, frame, formulate, generate, reflect, facilitate^60–63^. Systemic design has roots in human-centred or service design practice which center user (those most affected by a challenge) experiences; however, it significantly differs from these modes of design in that it holds deeper attention to mindsets, values, context, power relationships, leadership, and systems, and then connects these activities in a strategic learning system^64–66^. Recent critique of the field and practice of design from an equity and justice point of view is resulting in theory, frameworks, and approaches to encourage/enable/require centering equity in design processes more skillfully and consistently^67–70^.

Social innovation, systemic design, and decolonizing methodologies have strengths that were woven together into a different planning and policy making process in this applied research. In the next section we describe how we designed and implemented this weaving in our project, as well as into a framework that describes a transformative approach to policy making and planning processes.

## Results

### Experimenting with transformative process design and facilitation

**Fig. 1:**
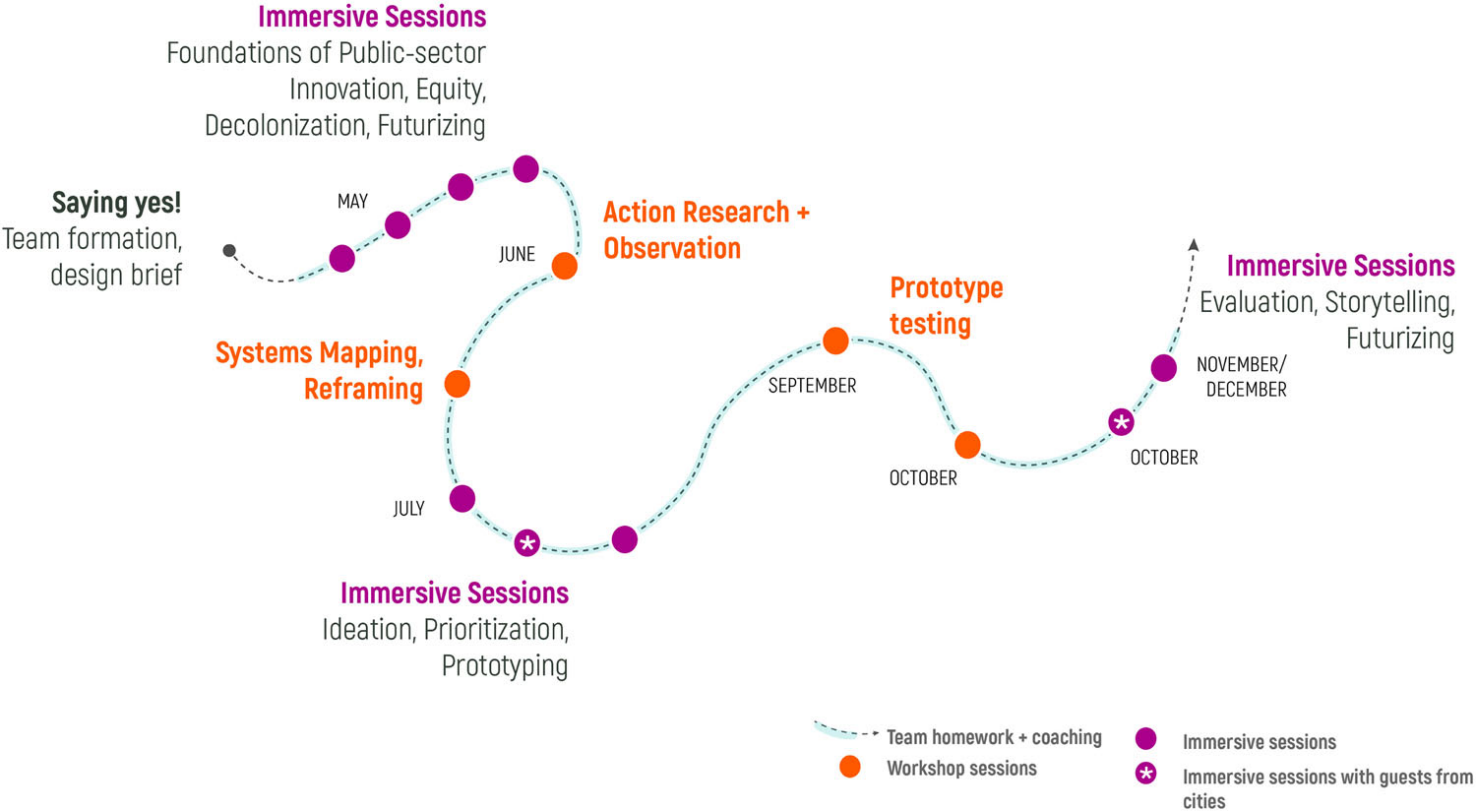
Transforming Cities from Within learning journey process map.

The transformative policy making and planning process framework described in this section resulted from a year-long participatory action research study (‘learning journey’) that was delivered in 2021. The learning journey was designed and facilitated by a diverse seven-person core research team (including the authors of this article), consisting of formal academic researchers as well as researcher-practitioners. Learning journey-goers, also known as our co-researchers, included municipal staff from multiple departments working in ten Canadian city-based teams (two of these teams included participants from community-based organizations), and staff from two Canadian network serving organizations (see Methods section for further details). Each team began their learning journey by preparing a design brief that described their understanding of the complex climate, equity, and decolonization question that they were beginning with, the North and Near stars (i.e. vision and goals) for their work, the theory of change informing their current approach to transformation, description of the people/places most impacted by their challenge, and the inner work and learning they committed to do. The learning journey was delivered through thirteen half-day virtual workshops (represented by the dots along the line) over nine months and active ‘invitations to practice’ (i.e. homework, represented by the connecting line) in between sessions. A visual representation of the process arc and high-level focus of each of the workshops and stages is shown in Fig. 1.

The learning journey was designed and facilitated as an intentionally transformative alternative to more standard and familiar processes. Each of the dots represents a half day synchronous, virtual workshop led by the core research team. Each workshop had a specific focus topic and practice, and overall the sessions were designed with consistent core elements, including: slow and reflective experience to enable deeper systemic work; mindfulness/embodiment practice; reflective practices; more formal teaching and content-giving; stories from the field; connecting and relationship building; team working/practice activities; and sharing our works in process. In between the workshops (the dotted line) were specific activities/homework, described as ‘invitations to practice,’ for the teams to apply and experiment with. The core research team used interactive slide decks, online collaboration tools and templates, one-on-one and team coaching with the core research team, and dialogue as central andragogical approaches. The teams experienced a transformative approach to policy making and planning which required them to reimagine and reframe their work. The process was designed to open up thinking about potential planning and policy making process interventions outside of what they were practiced/expert in, and to inquire into and challenge the paradigms that hold the current dominant ways of working in place as standard practice. It was also designed to carefully and lovingly hold the discomfort, tensions, and ambiguity that this approach generated for people to help them to stay with the learning journey.

### Transformative policy-making and planning process framework

**Fig. 2:**
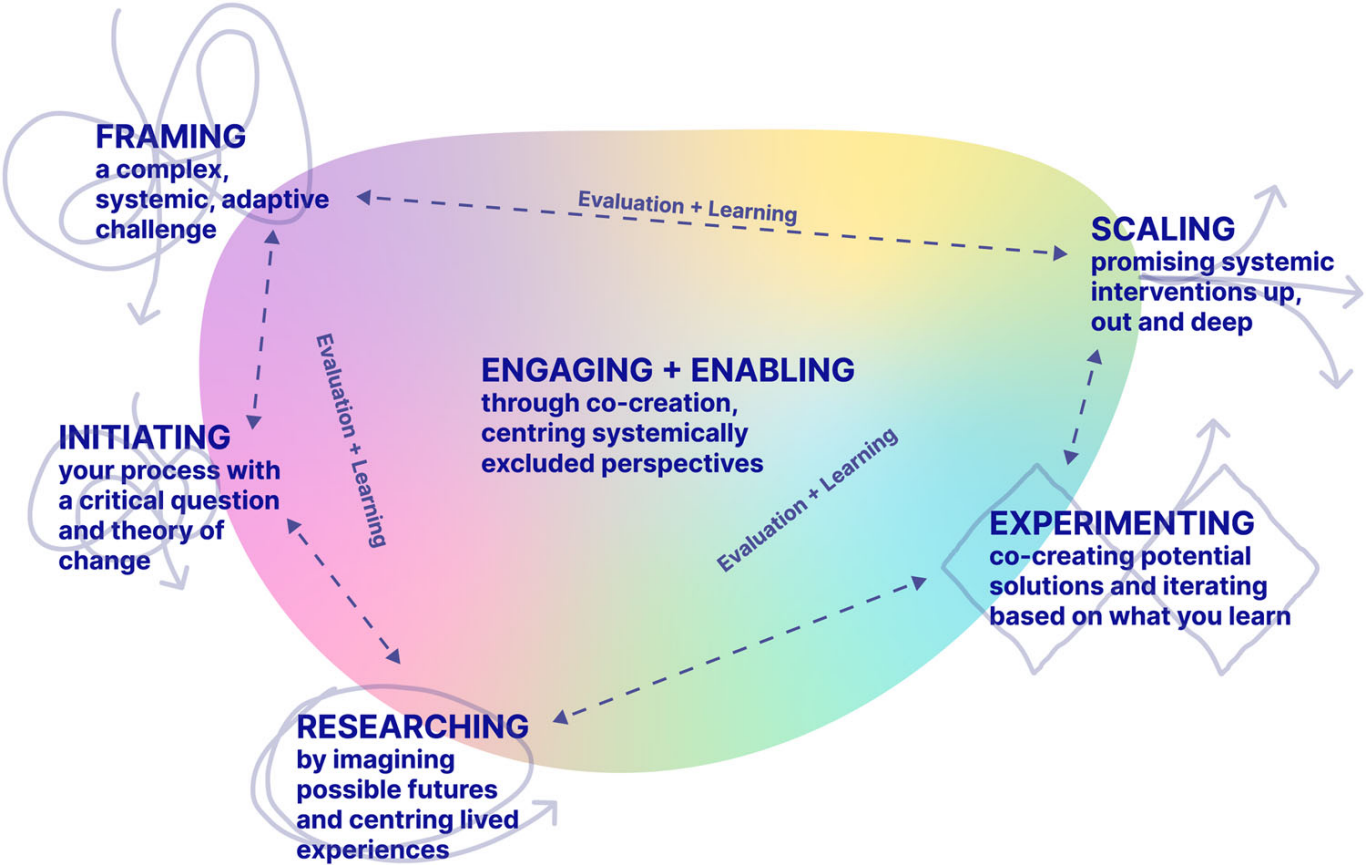
Transformative policy-making and planning process map.

This framework for transformative policy making and planning was generated through engagement with literature and through interacting with empirical data generated during the learning journey. This framework is presented here as two parts - a process map (Fig. 2), followed by a description of the transformative approaches taken at each stage of the process (Tables 1–6).

The process map captures key stages of this transformative approach, as well as the movement and connections between the stages. Note that these are not drawn as discrete, linear steps but rather as interrelated and entangled stages with iteration between. There is a general movement (indicated by the looping solid lines) from broad/messy/open/exploratory that begins with framing, and toward a gradual focusing/clarifying process as we move through initiating, researching, experimenting, and eventual implementation at the scaling stage. Engaging and enabling, and evaluation and learning, happen throughout. As the connecting dotted lines indicate, there is iterative movement between stages when insights and learning require/indicate reframing, renewed experimentation, and/or return to learning from those most impacted by the challenge.

### Transformative policy-making and planning approaches

Tables 1–6 detail the process map into descriptions of transformative approaches at each stage. Common policy-making and planning approaches are compared to transformative approaches at each stage, along with a description of how the transformative approach took shape in the learning journey. Some additional thinkers informed the ideas about the transformation that inspired these approaches^71–81^.

**Table 1:** Framing Stage

Common approach	Transformative approach	Brief description of how this transformative approach shapes process design
Climate, equity, decolonization are discrete	Climate, equity, and decolonization are interrelated	These complex challenges have related root causes (i.e. colonialism, capitalism, racism, structural oppression) and they should be addressed together to have systemic impacts. Example practice: the initial framing of a climate action project integrates relevant background information and context about how this issue impacts equity, justice, and reconciliation and fully integrates this into project framing rather than treating these as separate topics.
Planning is neutral	Planning is personal and rooted in place	Intersecting positionality, power, and privilege affect how work is framed. The construct of ‘neutral’ reinforces dominant colonial paradigms and practices. Work in relationships with Indigenous Peoples and place by building trust, recognizing the legitimacy of Indigenous knowledges, and understanding past and ongoing roles of urban planning and policy making in dispossession, oppression, and marginalization. Example practice: self-in-system mapping that articulates the intersectional identities, privileges, positionality, and power of members of the project team, with discussion about how this will impact the perspectives and biases that frame what is/n’t important and relevant in project framing.
Time is scarce and constrained	Time is abundant and non-linear	Make time for (re)framing complex challenges, relational ways of working, staying with ambiguity, and intervening at the root causes of deep, and systemic challenges. Example practice: project managers design for extra time and flexibility to respond to unanticipated process changes, and senior decision-makers enable this flexibility.
Challenges are complicated and technical	Challenges are complex, systemic, and adaptive	Complex, systemic, and adaptive challenges demand different approaches to working on them - they are unknowable, unpredictable, and emergent. These features require different approaches to learning and working with/on these types of challenges. Example practice: systems mapping is used to understand dynamics at work in the challenge, and to expand the view to include interrelated issues. Leverage point analysis is used to identify high impact places to intervene in the system through policy and planning work.

**Table 2:** Initiating Stage

Common approach	Transformative approach	Brief description of how this transformative approach shapes process design
Begin with outcome	Begin with question	Transformative processes begin with an active, relevant, and critical question that is (re)framed along the way as user research, experimentation, and learning reveals insights and possibilities. Example practice: designers often begin work with a creative question framed as a ‘How might we…’ or ‘What if…’ possibility. Good creative questions bound the challenge space and also open up creative possibilities and visionary potential.
Project charter and project management plan	Design brief and theory of change	Captures the vision, goals, values, process, evaluation and learning, and potential outcomes of an innovation process in a concise and compelling way. Outlines a structure for a process in a way that also allows for non-linearity, not knowing the outcome, taking a user-centered orientation, having a long-term and shorter-term vision and goals, and enabling creativity, experimentation, and learning. Clearly describes how a team thinks that change happens, and their intended/desired contributions to change. Example practice: a theory of change articulates vision, goals, a description of how a team thinks that change happens (in a theory-informed way), and how their work will contribute to that change. It is a helpful practice to surface implicit assumptions and uncover differences or tensions, and then to design for these in a productive way.

**Table 3:** Research Stage

Common approach	Transformative approach	Brief description of how this transformative approach shapes process design
Scenario planning	Speculative and visionary fiction	Imagines possible futures beyond the limited frames that planning and policy making typically take, by tapping into creative, speculative, protopic, future world-building through imaginative writing. Example practice: creative writing/drawing and worldbuilding using techniques from fiction can help a team to expand their sense of what is possible and necessary, to see potential pathways, and to be inspired by one another’s visions.
Policy research and best practices	Action and user research	Centres the lived experiences of people most affected by a complex challenge, the lived experiences of land, water, and more-than-human kin, and future generations in research. Example practice: ethnographic and/or action research insights are regularly returned to users to test how they are informing a planning and policy making process throughout in generative and reciprocal (rather than extractive) ways.

**Table 4:** Engaging and Enabling Stage

Common approach	Transformative approach	Brief description of how this transformative approach shapes process design
Inform, consult and engage	Co-Create	People and places most affected by a complex challenge are involved as co-creators in the process, in ways that work for them. Power and decision-making are shared. Accountabilities for implementation rest in multiple people and organizations. Example practice: bring together people holding diverse experiences and perspectives in a creative ideation/brainstorming process to generate possible solutions to the challenge at hand. Work together to prioritize the most promising possibilities based on shared prioritization criteria.
Distinct, hierarchical, disciplinary roles	Teams are self-organizing, whole, purposeful	Work of people in these processes centers on their skills, talents, and interests; hierarchical roles are left at the door. Creative ways to engage/work with and/or reject/work around the dominant, hierarchical structures are often required. Whole selves invited. Example practice: design a team structure that supports and enables distributed leadership amongst team members based on their areas of expertise, rather than within a hierarchical structure.
Stakeholders represent known interest groups	Center systemically excluded perspectives	Move at the speed of trust. Prioritize relationship building. Give this the time and commitment necessary, and be accountable to what is shared. Do not place a significant/unequal burden on staff who also have lived experiences of these perspectives to do this work. Example practice: design processes to make involvement by those typically left out possible, relevant, important, and culturally and emotionally safe. This might look like paying people for their time and expertise, providing child care or transportation subsidies, and designing multiple a/synchronous ways to provide input and feedback to maximize accessibility.

**Table 5:** Implementing Stage

Common approach	Transformative approach	Brief description of how this transformative approach shapes process design
Pre-determined solution	Experimentation and prototyping	Process assumes that we don’t know the ‘right’ answer, and that perhaps it will remain unknowable due to its complexity. In order to gain insights into fruitful, systemic directions to move in we must experiment, probe, learn. Example practice: design and implement small, low-fidelity, low risk, and cheap experiments to see if the ideas that are being generated are promising when tested in the real world, with real users.
Linear cause/ effect pathway	Iterate	As we experiment, we (re)connect to users, learn, and reframe the complex challenge, we do this in iterative cycles of increasing quality, fidelity, and systemic impact. Example practice: design an evaluation system to make sense and meaning of what is being learned through prototyping, and iterate (and throw things out) often. Pay attention to the patterns of learning happening across multiple prototypes, and what this is telling you about the larger system you are trying to shift through your intervention.
Project completed when plan/ policy approved	Project ongoing into implementation	Planning and policy-making processes are not distinct from implementation processes. All of the relationships, research, experimentation, learning, and insights continue into implementation, with a goal of scaling up/out/deep the successful systemic interventions into policies, programs, services, etc. Example practice: apply many of these approaches in a renewed way as you move into implementation of the policy or plan; transformative approaches need to continue on into implementation.

**Table 6:** Evaluating and Learning Stage

Common approach	Transformative approach	Brief description of how this transformative approach shapes process design
Council Report	Storytelling	Regrounds in purpose, people, possibility, and process. Reporting has multiple accountabilities in this type of process, and the form should reflect that. Enables creative, humane, complex, imaginative, and non-linear ways to talk about what happened/what might happen. Example practice: the communication materials and plan should be designed as a story with a compelling arc, an impactful expression of why this work matters and what’s at stake, and a clear call to action. Visuals (i.e. documents, slide decks) should skillfully enhance storytelling impact.
Quantitative/ summative evaluation at end of project	Learning, reflection, evaluation throughout	Evaluation happens throughout a process as a way to learn and reflect, document, and adapt the approach in real time based on the insights being generated. Evaluation has a strong user-orientation: who needs what information, for what purpose, and on what time scale to inform the kinds of decisions that they need to be making along the way. Example practice: developmental evaluation uses cycles of asking ‘what’, ‘so what’, and ‘now what’ throughout a process, drawing upon multiple intelligences and perspectives of people involved, and then adapting/iterating the process in response.

This transformative policy-making and planning framework can be considered as a whole, and can also be considered as smaller/independent interventions into unfreezing standard processes when an overall process redesign is not possible or supported. Careful creation/curation of transformative processes that centre learning are essential when working with practitioners who are used to being expert professionals in standard policy-making and planning processes, with many thoughtful, intentional, and reflective process design choices required to do this well. The next section describes seven of these significant choices and interventions from our research as key moves to provide texture about how policymakers and planners might think about designing and delivering a transformative process.

## Discussion

The seven key moves toward transformative policy-making and planning processes explained below were intentionally integrated into the learning journey as an attempt to experiment with meaningful entry points into decolonization practices for planners and policymakers. The hope was (and is) that participants might enact these moves in their everyday professional lives. We express these practices as moves (rather than steps) because significant reflection and interaction took place during and between each session for both learning journey goers and the research/facilitation team. For example, the research team/facilitators had to consistently design for/work with/be comfortable with emergence and ambiguity, as the learning journey often and intentionally challenged participants to turn inward, reflect on their identities and positionality, show up as their authenticity selves and encouraged vulnerability in small and large group settings. The details of these moves are discussed below.

### Move 1: beginning well

Our core research team members each had previous experience with designing and delivering transformative processes in diverse public sector, community-based, and academic environments which gave our team a strong collective foundation from which to grow. We began by crafting a compelling invitation to join this process based on the real-world challenges that we knew city staff were struggling with. This clarity of invitation and sensing of the field meant that the right people found their way in, and we had very little attrition along the way. The learning journey started with an intensive virtual retreat in order to co-create excitement, build relationships, and establish clarity about what people wanted the journey to be for them. The retreat series included connecting to land, place, history, context, and Indigenous pasts/presents of each place where cohort members were coming from. One cohort member reflected that **“**I think one thing that was probably challenging for us at the beginning was figuring out how these things relate to engineering and our very specific transportation engineering field.*”* The retreat series also included cohort members reflecting on their personal and team calls to action and accountability based on their identities and experiences, reframing the complex challenges that they were coming in with based on systems practice, and imagining possible and desired future states resulting from their transformative work. These choices clearly signaled that this process would be different from other planning, policy-making, and/or professional development processes than they were used to.

### Move 2: co-creating an equitable, just, feminist, and decolonized space

Concepts of equity (e.g. power, privilege, anti-racist approaches) were intentionally (re)introduced throughout the learning journey to practice foundational concepts and develop a shared language. Grounding the process in place-based, relational, and decolonial approaches set the tone for how the cohort was invited to bring themselves into the space. Some features included the practice of abundant time, and the dance between this and the sense of urgency associated with the nature of the challenges everyone was working on. We did deep self-in-system work to explore how our individual positionality, intersecting identities, and life experiences impact how we each see, understand, and experience the world and the challenges we were working on. We worked to transform the construct of being ‘expert’, and of ‘professional’ looking and behaving in a particular way characterized by colonial and white dominant paradigms. One cohort member described it this way: “The reimagining of these core city processes has the potential to impact other systemic challenges as well, because at its foundation this is a shift toward more place-based, people-centered, experimental, and exploratory processes where we collectively need to lean into the emergent solutions that we are working toward because they are not yet known.” Ongoing reflection on how there is no step-by-step guide to do decolonial practice was central in the process as, for most of us, this is a lifelong learning process filled with discomfort.

### Move 3: pro-love pedagogy

Partway through the process our research team began using the phrase pro-love pedagogy as a short-form descriptor for a collection of choices we were making in design and facilitation. Pro-love meant that we prioritized being in a caring, loving, supportive, and collaborative community with/for each other as research team members, and consequently how the cohort experienced the holding space that we co-created with them throughout the process^75,81^. One member of our research team reflected that “being in a caring and loving research team changed my perspective about research, and what that can look and feel like.” Evidence of this pro-love pedagogy looked like an ongoing trust and willingness of the cohort to come along on this journey, even when it got uncomfortable and they didn’t know where it was taking them. It looked like the research team navigating and holding our own and each others’ discomfort, dis-ease, and uncertainty and modeling a skillful and honest navigation of this liminal space. It looked like an opening up to the diverse experiences and perspectives of the research team to co-create a rich learning-oriented process for everyone even when it wasn’t clear how all of the pieces fit together. It looked like radically trusting ourselves and each other at every step along the way.

### Move 4: focus on practicing not problem solving

The process emphasized building and practicing competencies and capacities for transformation rather than on solving problems. We thought about this as exercising different muscles, and rehearsing the new/resurgent. We did this by co-creating a learning, practice, experimentation, and reflection-focused space with/for each other. The dominant problem-solving orientation in planning and policy-making processes taps into well-honed and exercised skills, habits, approaches, and subsequent results. We needed the cohort to question, reframe for themselves, and look beyond this standard approach if they were to have a real chance at getting to a different outcome. Working on complex, ambiguous challenges demands a different set of skills and approaches, and practitioners can quickly find this overwhelming and daunting to navigate. One cohort member shared that the journey helped them to “think about how we can overlap our different programs to create multiple effects instead of just using one program to treat one problem.”

### Move 5: drawing on different theories and practices

Our research team carefully curated a set of theories, approaches, and practices that are not commonly used in city governments, including action and user research, systems mapping, prototyping, reflective practice, developmental evaluation, imagining possible futures/speculative fiction, prototyping, and others. The cohort worked the techniques, made mistakes and had breakthroughs, shared their experiences with one another, and built their literacy and confidence in approaches more suited to the complex challenges they were working on than their go-to approaches. An example of this was the regular returning to systems mapping throughout the journey to develop each person’s understanding of the deeply connected and shared roots of climate, equity, and decolonization challenges. We worked and iterated these systems maps as people expanded and shifted their perspectives, and then used the maps to generate promising fractals and feedback loops where they could intervene. One participant captured their experience in this way: “It’s so easy to just be in the normal ways of doing and being; it’s so ingrained and easy to go along with it. I realized I can question this, and that it takes so much presence and intention to hold a different possibility as this isn't part of people’s jobs or recognized as legitimate work.”

### Move 6: working with fear

We also noticed the implicit influence of fear as a motivator of in/action and behaviour - fear of messing up, of saying the wrong thing, of offending, of failing to have impact. Surfacing and shining a light on these fears and supporting the cohort to develop different, more generative relationships with these fears and what they might contribute or catalyze was an important part of the process. A particular fear that many people acknowledged was that they lacked the skills, abilities, and connections necessary to work in highly relational, respectful, and mutually beneficial ways with the people and communities whom they serve, and who are most impacted by these intersecting challenges. One cohort member said that they “don't have the relationships with equity-denied people and groups. The fear that staff have is projected onto people - otherizes them, focuses on risks, and is based in fear. What if the foundations of our work were about good relationships instead?” Another member realized that we need to “prioritize the time and space needed to reframe the complex problem, asking ‘who have we not talked to?’ and then prioritize action and user research to understand their experiences and perspectives.”

### Move 7: redefining impact and outcomes

Standard planning and policy-making processes offer the comfort of a clear endpoint - the successful approval of a piece of work by decision-makers, elected officials, and community stakeholders. Transformative processes are not as clear and tidy. They are concerned with transformation at the personal, team, organizational, cultural, relational, and systems scales and in this case, on complex intersecting climate, equity, and decolonization challenges. There is no clear finish line in this work as it is long-term, ongoing, and generational. Cycles of action, learning, and reflection are necessary, putting planning and policy-making processes in a direct and ongoing relationship with implementation, rather than viewing them as separate processes. Generating other ways to measure, understand, reflect upon, evaluate, and tell stories of impact was an important part of grounding the experience of this process for people, and supporting them in articulating why it mattered and what resulted. We used ongoing developmental evaluation, speculative fiction, and persuasive storytelling to support individual and collective sensemaking about impacts.

Imagining, enabling, and enacting transformative policy-making and planning processes will require equipping both new and established planners and policymakers with emerging/resurging approaches to complex urban climate, equity, and decolonization challenges. The transformative policy-making and planning process framework shared here intends to support research and practice in this important domain, and contribute to enhancing research and action more generally on complex, intersectional urban sustainability challenges.

## Methods

### Participatory action research bricolage

The core method used in this study was participatory action research (PAR), defined as “a participatory process concerned with developing practical knowing in the pursuit of worthwhile human purposes. It seeks to bring together action and reflection, theory and practice, in participation with others, in the pursuit of practical solutions to issues of pressing concern to people, and more generally the flourishing of individual persons and their communities” (p.4)^82^. In PAR, research subjects are co-participants in the processes of inquiry which includes activities of generating questions and objectives, sharing knowledge, building research skills, interpreting findings, and implementing and measuring results. Recent turns in PAR call for the method to support transformation while providing rigour and quality in ethics, process, and outcomes and this provided additional guidance to our approach^82–84^. Bradbury et al. articulate seven choice points for quality action research as: (1) articulation of objectives; (2) partnership and participation; (3) contribution to action research theory/practice; (4) methods and process; (5) actionability; (6) reflexivity; and (7) significance which informed the method taken in this work^82^.

PAR was embedded within an interdisciplinary, critical, qualitative, activist, and practice-oriented bricolage^85,86^ “concerned with the depth and complexity of a question, what lies below the surface, and the form in which that complexity might best be understood and revealed” (p.2)^87^. This methodological layer was added to ensure that PAR would critically engage with the dominant systems and structures of planning and policy-making that this research was aiming to question and reimagine. The two authors of this paper, along with the five additional members of the core research team, each brought diverse lived experiences, professional practices, distinct disciplinary lineages, and diverse positionalities and perspectives together to construct this PAR bricolage, and to design, facilitate, and generate insights about this learning journey together based on our collective of experiences and perspectives. Indigenous, feminist, anti-oppressive, and ecological methods were features of our bricolage, and necessary to better support working in/with processes of transformation, and to find our ways out of the dominant paradigms of planning and policy-making through our methodological approach^88–97^. Using a PAR bricolage meant that we took an orientation of gathering up and applying what was useful from these different methods, in a generative dialogue with these methods and with each other. This also helped us to remain focused on being in service to generating insights about our practice-oriented questions, and those of our 40 co-researchers in the learning journey. This methodological approach enabled our research team to balance theoretical rigour, transdisciplinarity, practice-based foundations, and emergence. Together we held an entangled and engaged researcher orientation where the opinions, perspectives, and experiences of our research team and action co-researchers were considered a strength.

### Research design

The University of British Columbia Behavioural Research Ethics Board provided approval for this study (H17–02282). All research participants provided ‘written informed consent’ to take part in the study. The core research team crafted the learning journey and invited participation through an open call that was distributed through existing networks supporting city governments working on climate and equity-related work in Canada. This invitation was open to public sector staff working within City governments, and could include team members from community-based partner organizations if this would be relevant to the challenge that the team wished to work on during the journey. It was also open to network-serving organizations that worked to support City governments in their climate, equity, reconciliation, and/or decolonization work. All of the ten Canadian city-based teams (two of which had community partners) and two network-serving organizations that applied to participate in the journey were invited to join. City staff were from the environment, sustainability, social policy, planning, engineering, equity and reconciliation, and public engagement departments/roles. Teams completed the application requirements, and our research team held the approach of saying yes to as many teams that were ready as we could properly support. One team dropped out of the learning journey about halfway through due to their limited capacity to fully participate, although they continued to be invited into all learning journey activities through to the end of the project. Team’s were informed that this was an applied research project before applying, and upon acceptance into the learning journey their formal consent to participate was confirmed. Several new people joined the journey part-way through, with their consent confirmed when they joined.

Each multi-departmental team brought a different, locally relevant, complex planning and/or policy-making climate, equity, and decolonization challenge that they actively worked on throughout the nine-month-long learning journey held from April - December 2021. Social innovation, systemic design, and decolonization theories, processes, and practices were used to design the backbone of the learning journey. Other theories and processes informed the andragogical approach as well, including transformative adult learning; critical race theory; feminist and queer methodologies; sustainability transitions; complexity and emergence; and AfroFuturism. Our core research team shared the responsibility for the overall design of the learning journey, with individual team members and external guests invited in to lead and facilitate specific workshops based on their expertise and experience. The research team engaged in ongoing reflective practice throughout the whole journey, documenting insights and learning along the way, and shifting and pivoting the design of the journey in response to what was happening in-session with co-researchers and their emerging questions and needs.

Qualitative data was collected throughout the journey, and included observation, interviews, reflection of the research team and cohort members, team coaching sessions, document review, active process design and facilitation interventions and their impacts, and shared sense-making with the research team to uncover themes and insights. Active and ongoing sensemaking of this data occurred through regular dialogue amongst research team members, tracking what was emergent, responding through the design of the learning journey, and noting themes throughout. Emerging insights and themes were regularly returned to the co-researchers during the learning journey workshops to ensure that the sensemaking being done by the core research team was reflective of the co-researchers’ experiences. Active consent of cohort members to participate in the research continued throughout the journey, including reaffirmed consent at their last interview three months after the journey had finished.

### Reporting summary

Further information on research design is available in the Nature Research Reporting Summary linked to this article.

### Supplementary information

Reporting Summary

## Data Availability

Data from this research is not available for other users or purposes, as per our behavioural ethics board review and approval.
